# Comparison of admission rates among patients treated by male and female emergency physicians: a multicenter study

**DOI:** 10.1186/s12873-020-00349-4

**Published:** 2020-07-01

**Authors:** Hisham Valiuddin, Hope Ring, Michelle Fallon, Yaser Valiuddin

**Affiliations:** 1 Department of Emergency Medicine, St. Mary Mercy Hospital, 36475 Five Mile, Livonia, MI 48154 USA; 2 Edward Via College of Osteopathic Medicine, 2265 Kraft Drive, Blacksburg, VA 24060 USA

**Keywords:** Gender, Admission rate, Male, Female, Emergency physician, Admit

## Abstract

**Background:**

No study to date has looked at the gender of emergency medicine (EM) physicians in the United States in relation to admission rates. This study seeks to investigate admission rates of adult patients treated by female vs male EM physicians, to identify whether a practice pattern bias exists.

**Methods:**

This was a multicenter retrospective study of four community hospitals. Population: All patient encounters between July 1, 2016 and June 30, 2017. Outcome: We compared multiple benchmarks, including admission rates, patient acuity, length of stay, return visits, patient age, and years of practice using descriptive statistics and Pearson Correlation Coefficients.

**Results:**

171,762 encounters by 71 EM physicians; 29 females, 42 males. Average admission rates: female 30.1%, male 28.0%, *p* = .188. Average encounters: female 2456, male 2394, *p* = 0.77. Acuity: female 149.3, male 146.9, *p* = .227. Average length of stay (minutes): female 294.4, male 277.4, *p* = .137. Average patient age: female 50.9, male 50.2, *p* = .457. Median time of encounter: female 12.8, male 12.7, *p* = .964. Years of practice: female 16.2, male 19.1, *p* = .274. Average return visits per one thousand: female 8.5, male 8.5, *p* = .864. Secondary analysis of Pearson Correlation Coefficient of Significance; admission rate and length of stay: female 0.53, *p* = .0026; male 0.76, *p* < .0001. Admission rate and acuity: female 0.56, *p* = .0012; male 0.76, p < .0001. Admission rate and patient age: female 0.54, *p* = 0.0018; male 0.50, *p* = 0.0003.

**Conclusion:**

No statistically significant difference exists between the admission rates of male and female emergency medicine physicians. The admission rate in both groups had the highest correlation with patients’ age, acuity, and length of stay.

## Background

Throughout every shift, an Emergency Medicine physician is making a plethora of decisions ranging from what pain medication to give a patient to whether a lifesaving intervention needs to be performed. Arguably one of the most important decisions a physician makes each shift is whether that patient needs to be admitted (inpatient or observation) or whether that patient is safe for discharge. A myriad of factors influence this decision, including but not limited to the patient’s age, comorbid conditions, the support system at home, access to care, and the acuity of their condition. One study in Australia found strong evidence to suggest that personal sociodemographic and health characteristics are major drivers of preventable hospitalizations [1]. While another study found that avoidable hospitalization rates are lower in areas with more primary care physicians-per-person according to bounded-area density measures [2]. Factors such as emergency department volume, time of patient presentation, and levels of senior support were also identified as non-clinical influences on the decision to admit rather than discharge patients [3]. We ask the question, does the gender of the treating physician also influence this decision?

Multiple studies across other specialties have noted that females and males can practice medicine differently. Females currently make up 50% of medical school graduates and 25% of the Emergency Physician workforce [4]. Studies in Internal Medicine have looked at patient outcomes as a function of physician gender, finding that elderly patients treated by female internists had lower mortality and lower readmissions [5]. Another article reported that patients operated on by female surgeons had a statistically significant decrease in 30-day mortality [6]. One explanation for this difference was that female physicians are more likely to follow evidence-based guidelines [7]. It has also been noted in the literature that females employ a more “patient-centered” approach to patient care and engage in more preventative services for their patients than their male counterparts. Due to their communication style, patients of female physicians tend to disclose more biomedical information to them as well [8]. However, given these positive findings, there still exists a negative gender bias against female physicians in the workplace [9]. An example of such is a facility in Ontario that recently faced allegations for discriminatory hiring practices when it was found out that no female emergency physician was hired at the ED in 16 years [10]. Even after being hired, there exists gender disparity. Numerous papers have noted that female physicians are paid less than their male colleagues. Depending on the specialty, the gender pay gap can be anywhere from 16 to 37% [9]. This study seeks to provide evidence for dismissing such discrimination and could be used to further the arguments for gender equality and equal pay by demonstrating that no practice pattern bias exists based on gender alone.

No study to date has investigated practice patterns by analyzing a possible correlation between admission rates of adults in the US and the gender of treating emergency medicine physicians. Our study aims to discover whether there is an inherent gender bias in the decision to admit a patient with our hypothesis being that there will be no association between gender and admission rate.

## Methods

We conducted a retrospective study analyzing all patient encounters from four hospitals over the July 1, 2016 to June 30, 2017 academic year. This study was approved by the Institutional Review Board with a waiver of consent.

This study was performed at four community-based hospitals that represent a varied patient population. The study institution and sister hospitals within the same health care system in southeast Michigan were selected based on the inclusion criteria of having more than one hundred inpatient beds and more than fifteen thousand ED visits per year. Primarily pediatric institutions were excluded. Trauma levels for each ED were certified by the American College of Surgeons. Geriatric emergency department designation was certified by the American College of Emergency Physicians. (Table 1).

**Table 1:** Characteristics of Study Sites in 2016–2017

Hospital	Hospital #1	Hospital #2	Hospital #3	Hospital #4
Trauma Designation	Level 2	Level 1	No designation	No designation
Geriatric Designation	Level 1	No designation	No designation	No designation
Total ED Beds	51	65	19	20
Total Hospital Beds	304	537	136	133
ED volume (per year)	52,000	88,000	24,000	18,000
Residents or APP^a^ present	Yes	Yes	Yes	Yes

^a^*APP* Advanced practice providers (nurse practitioner or physician assistant)

Each ED was always staffed by emergency medicine board-certified or board-eligible physicians. All facilities were supported by residents or advanced practice providers. Advanced practice providers were defined as nurse practitioners or physician assistants.

**Fig. 1:**
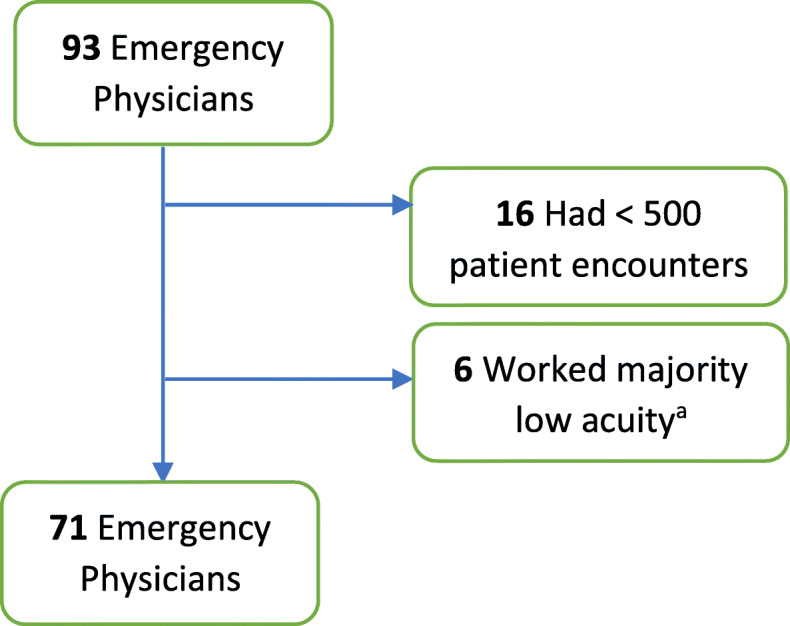
Physician inclusion – exclusion criteria. ^a^ Worked majority of shifts in low acuity section of department, seeing mostly low acuity triaged patients

Data was collected on emergency physicians who had at least 500 patient encounters per annum. Any patient encounter that resulted in a transfer or an elopement was excluded. Patient encounters for mental health diagnosis as determined by the *International Classification of Diseases, Ninth Revision, Clinical Modification* [ICD-9-CM] codes were also excluded, as the disposition plan for these patients was predicated upon strong discretion from the consulting psychiatric social worker evaluation and the resource availability at each separate study institution [11]. Physicians who worked majority in “fast-track” shifts, dedicated to seeing only the lowest acuity triaged patients (Emergency Severity Index 4–5) were excluded (Fig. 1) [12]. Using a data analytics registry we compiled physician metrics, benchmarks, and demographics; recording the admission rates, number of years of practice of physicians, average patient acuity treated by physicians, length of stay (LOS), patients age, number of encounters, number of male patients treated, number of female patients treated, time of encounters and total return visits after discharge from ED in 72 h.

We then grouped the physician study population into male and female categories. Using descriptive statistics, we analyzed both groups. A secondary analysis was also performed to identify the interrelation of variables.

After the hospitals of interest were selected, we extracted data using an abstraction tool (Additional file 1), categorizing entries by physicians. The average acuity of patients seen by each physician was calculated based on the level of coding of the chart and associated critical care time billed. The level of coding was determined by billing experts from a revenue cycle and practice management company with greater than 30 years of experience, responsible for billing more than one hundred institutions, by reviewing physician charts and incorporating procedural codes. We weighted each level of coding and associated critical care time per the Medicare reimbursement schedule to calculate a composite score, which served as the average level of acuity of per patient seen by that physician [13]. To illustrate further, if a hypothetical physician only saw the lowest acuity patients, such as visits for suture removal, the physician would have a calculated composite score of 23; in contrast, if a hypothetical physician only saw the highest acuity patients, such as acute respiratory failure, the physician would have a resultant composite score of 236.

The average length of stay was recorded in minutes per patient encounter. Time was started upon triage registration of the patient and ended when the determined disposition order was placed. Demographics of patient encounters included average patient age at the time of encounter and gender of patients treated. Benchmark data included average return visits per one thousand patient encounters and the total number of patients encounters over the study period per physician. Ancillary information such as years of practice and time of encounter were also recorded and assessed to determine if these variables were potential confounders. Years of practice were calculated by years removed from the date of graduation of medical school. Time of encounter was recorded using a military time scale and a median time of encounter was determined for each physician. Physician data, including physician age, was aggregated by querying Doximity, a comprehensive online database of US physicians that assembles networking services for US physicians through data partnerships, including the American Board of Medical Specialties, state licensing boards, and collaborating hospitals and medical schools. Each physician was then classified as either male or female. After database finalization, unadjusted differences between genders were compared using t-tests and chi-square tests, depending on the variable distribution. The t-tests were performed using the Satterthwaite adjustment for unequal variances. Adjusted estimates were not performed, as no significant gender differences emerged on the possible confounding variables.

The primary outcome was to assess differences in admission rates between male and female emergency physicians. The admission rate is defined as the portion of encounters that resulted in admission to the hospital. Admission includes patients who were dispositioned to an observation unit, inpatient unit, or emergency surgery. Secondary outcomes measured statistical differences between the two genders for the other study variables. The tertiary analysis was conducted to assess the correlation between average patient characteristics variables and admission rates at the physician level. As this was a tertiary analysis, no regression adjustments were performed.

The cut-off for statistical significance was set at *p* < 0.05 (two-tailed). The tertiary analysis was determined by comparing each variable with the admission rate using Pearson’s *r*. All analyses were performed in SAS statistical software (SAS, version 9.4, SAS Institute Inc., Cary, NC).

## Results

Overall, there were 171,762 ED patient encounters, treated by 71 total physicians, of whom, 29 (41%) were female and 42 (59%) male. The average admission rate for female physicians was 30.1% (range 19.3–43.8%) and male physicians had an average admission rate of 28.0% (range 14.3–40.6), which was not statistically significant (Table 2).

**Table 2:** Gender averages

Variables	Female (*n* = 30)	Male (*n* = 47)	T test (*p* - value)
Acuity^a^	149.3	146.9	0.227
Length of stay (minutes)	294.4	277.4	0.137
Return visits per 1000 encounters (patients)	8.4	8.5	0.864
Patient age (years)	50.9	50.2	0.457
Years of practice	16.2	19.1	0.274
Number of female patients (per annum)	1074	1051	0.804
Number of male patients (per annum)	1382	1343	0.752
Total patients (per annum)	2456	2394	0.770
Median time of encounter (24 h)	30.1	28.0	0.188

^a^Based on weighted average of billing codes per Medicare reimbursement (Range 23–236, with 236 as most critical)

Table 2 displays the remaining variables that were analyzed regarding the patient-physician encounters, as well as their respective *t*-tests. No statistically significant difference was found for any of the patient demographics. The acuity of patients seen, average patient encounter LOS, and the average number of return visits in 72 h per one thousand patient encounters were not statistically different for either group. Both physician groups had a similar composition of career experience in terms of years of practice and also were similar in the volume of patients seen. The gender breakdown of the patient census treated by both groups and median time of patient encounter was equivalent in both groups as well. The. These results suggest that males and females saw similar types of patients.

From all the variables that were examined, admission rates by both physician groups were found to have the strongest positive correlation with acuity level of patients: male 0.76, *p* < 0.0001 and female 0.56, *p* = 0.0012. Also, both groups had a strong positive correlation between admission rate and length of stay: male 0.76, *p* < 0.0001 and female 0.53, *p* = 0.0026. There was a moderate correlation in both groups between admission rate and the patient age: male 0.55, *p* = 0.0018, and female 0.50, *p* = 0.0003.

## Discussion

In this study, no gender practice pattern bias was found regarding the admission rate. This study is the first of its kind in the US. International data in different populations has suggested a possible correlation previously. One study conducted at a single pediatric ED in Canada, found no correlation, while another study conducted at a single adult ED in Spain found female physicians had a higher admission rate [14]. This is the only multi-center study on the topic and the largest study to date. Looking at many measurable factors and recordable characteristics, we found there to be no unadjusted statistical difference between the admission rate of female and male EM physicians, while the patients’ characteristics were similar between genders. This provides evidence that both groups make disposition decisions based on a patient’s clinical condition and situation. This can be noted by the Pearson correlation coefficients, which found a positive correlation in both groups between admission rates and acuity of patients, LOS, and patient age. Patients who were admitted to the hospital tended to be sicker, older in age, and have a longer LOS in the Emergency Department.

Our study sample analyzed data from four different institutions to assess a varied population of physicians and patient encounters. Previous literature, Miro et al., suggested that female emergency physicians admitted a statistically significant larger percentage of patients [15], differing from the results we found. One possible explanation is that Miro et al., was a single hospital study with only 50 providers enrolled. As with any study with small sample size, any discrepancy of a single physician could have profound effects on the study population. In studying a larger population of physicians, we found that the difference quoted in the prior study did not correlate with our results.

For the secondary outcomes analysis, we also recorded physician data on average patient return visits to investigate the possible correlation with the admission rate, which is novel to this study. Moreover, if a statistically significant difference in admission rate was found, there would have been an analogous difference in patient return visits. No such pattern was identified. Also, we found no gender disparity in the rate of return visits, as well as no correlation between the admission rate and return visit rate per gender group. When discussing return visits, caution is needed to interpret the results in the appropriate context. Many factors can account for a patient returning to the ED within 72 h of discharge; including expected reasons such as resource availability or need for serial exams. Average return visits are not explicit surrogate measures of quality of care delivered upon initial visit, and this secondary analysis further adds to the evidence [16–18]. Further studies are needed for a robust analysis of patient outcomes concerning admission rates and appropriate disposition planning.

Our overall finding of no association between gender and admission rate, as well as additional findings of no association between gender and volume of patients seen, acuity of patients treated, LOS, years of practice, time of encounters, or return visits, all add critical information to the conversation of evaluating gender disparities of practicing emergency medicine physicians. For instance, gender disparities have previously been reported among physician salaries. Watts et al. reported that between full-time EM faculty, females were paid 10 to 13% less than their male counterparts [19]. Madsen et al. validated this difference, reporting that women on average earned $19,000 less than their male colleagues, working a similar number of clinical hours; concluding that significant disparities continue to exist in representation, rank, and salaries of academic physicians [20]. Some of the reasons for this difference is theorized to be attributed to gender differences in negotiation, due to perceived conscious and unconscious biases, along with differences in opportunities to advance career and receive promotions [21, 22].

No practice pattern bias was identified in our study using objective measures, which further sheds light on gender studies of practicing emergency medicine physicians. Having this evidence, further studies can be conducted exploring gender disparities that are important in identifying unknown biases.

There are some limitations to this study. The sample of physicians studied were all employed by the same corporation, practicing within the same hospital system and geographic area. Consequently, practice patterns could be affected by hospital-wide protocols, clinical pathways, and guidelines. Although this limitation would similarly affect each group, making intergroup comparisons reliable, specific rates and characteristic information from this study may not be generalizable nationally. Second, the acuity of patients was determined using billing codes based on charting. Billing experts optimized the medical charts for the level of billing but cannot compensate for omitted data from medical charts resulting in a lower level of billing when in fact, acuity of the patient could be higher. Lastly, the gender of physicians and patients were recorded in categorical variables defined as female or male; therefore, no further investigation could be conducted about transgender or non-binary individuals.

## Conclusion

In summary, there is no statistically significant difference between the admission rates of male and female emergency medicine physicians. For both groups, the admission rate had the highest correlation with patients’ age, acuity, and length of stay.

## Supplementary information

**Additional file 1.**

## Data Availability

The datasets generated and/or analyzed during the current study are not publicly available due to individual privacy but are available from the corresponding author on reasonable request.

## Abbreviations

ED: Emergency Department
EM: Emergency Medicine
ICD-9-CM: International Classification of Diseases, Ninth Revision, Clinical Modification
LOS: Length of stay
SAS: Statistical Analysis Software
