# Supplementary material for: Comparison of admission rates among patients treated by male and female emergency physicians: a multicenter study
Source: BMC Emerg Med. 2020 Jul 1;20:54. doi: 10.1186/s12873-020-00349-4 (PMC7329465; doi:10.1186/s12873-020-00349-4)
Supplement: Supplementary file 1 — Additional file 1. [file 12873_2020_349_MOESM1_ESM.docx]

Abstraction Template: **Retrospective Study Data Collection Sheet**

|  | Provider | Admission | Acuity | Age | Total | Total | Years | Gender of | Return admits for | Timing of | Length |
| --- | --- | --- | --- | --- | --- | --- | --- | --- | --- | --- | --- |
|  | Name | Rate | Of Patients |  | Male Patients | Female Patients | Of Practice | Physician | total 1000 visits seen | Physician encounters | Of Stay |
|  |  |  |  |  |  |  |  |  |  |  |  |
| *Example 1* | Jane Smith | 15% | 130 | 75 | 200 | 180 | 4 | F | 4 | 0900 | 241 |
|  |  |  |  |  |  |  |  |  |  |  |  |
| *Example 2* | Joe Coffee | 40% | 200 | 71 | 600 | 601 | 8 | M | 10 | 1200 | 213 |
|  |  |  |  |  |  |  |  |  |  |  |  |
| *Example 3* | Clark Kent | 80% | 30.5 | 65 | 1700 | 1500 | 20 | M | 2 | 2100 | 194 |
|  |  |  |  |  |  |  |  |  |  |  |  |

| **LEGEND - Terms** |  |
| --- | --- |
| Admission Rate | Per annum for the Academic year of July 1, 2016 to July 1, 2017 |
| Acuity of patients | As calculated per weighted formula based on billing code compensation |
| Age | Average age of patients treated, at time of encounter, in years |
| Total Male Patients | # of male patients seen by provider |
| Total Female Patients | # of female patients seen by provider |
| Years of Practice | Years out of residency for treating EM physician |
| Gender of Physician | M (Male) or F (Female) |
| Return admits per 1000 patients | Number of patients that returned to ED after being discharged from ED |
| Timing of physician encounters | Median shift the physician worked on a scale of 2400 |
| Length of Stay | Average Length of stay of patient encounters in minutes |
